# Fulani show decreased susceptibility to *Plasmodium falciparum* infection *versus* Mossi: data from a community-wide screening and treatment of asymptomatic carriers in Burkina Faso

**DOI:** 10.1186/1475-2875-12-163

**Published:** 2013-05-16

**Authors:** Alfred B Tiono, Sodiomon B Sirima, Kamal Hamed

**Affiliations:** 1 Centre National de Recherche et de Formation sur le Paludisme, Ministère de la Santé, 01 BP 2208, Ouagadougou, Burkina Faso; 2 Novartis Pharmaceuticals Corporation, One Health Plaza, East Hanover, NJ 07936-1080, USA

**Keywords:** Malaria, *Plasmodium falciparum*, Mossi, Fulani, Artemether-lumefantrine

## Abstract

**Background:**

The Fulani ethnic group is known to have a lower susceptibility to *Plasmodium falciparum* infection than the Mossi.

**Methods:**

This commentary describes data from a recent cluster-randomized trial of community-wide screening and treatment of asymptomatic carriers of *P. falciparum* in 18 villages in Saponé, Burkina Faso.

**Results:**

The Fulani groups had a lower proportion of asymptomatic carriers at any occasion, a lower density of asexual forms and gametocytes of *P. falciparum* at baseline, and, in children under five years of age, lower rates of symptomatic malaria episodes per person-year than the Mossi.

**Discussion and conclusion:**

These data confirm previously reported differences in *P. falciparum* susceptibility between Fulani and Mossi.

## Background and methods

Recent investigations into the ethnic susceptibility of Fulani and Mossi for *Plasmodium falciparium* infection have consistently reported that Fulani have a lower susceptibility than Mossi [1-5]. A recent cluster-randomized trial in 18 villages in Saponé, Burkina Faso, which investigated the systematic, community-wide screening and treatment of asymptomatic carriers of *P. falciparum* with artemether-lumefantrine (AL) [6], provided further data on the susceptibility differences between these two ethnic groups. This trial enrolled a combined majority of Fulani and Mossi subjects and assessed the incidence of microscopy-confirmed asymptomatic carriage, asexual forms and gametocytes, as well as ‘symptomatic malaria episodes including fever and a parasite density >5,000/μl’ (abbreviated as SMRC5000 throughout the text) at various time points over the 12-month study [6].

While the exact biological reason for the lower susceptibility to *P. falciparum* of Fulani compared with Mossi is not fully understood, it is known that it is not due to an increased frequency of classic malaria-resistance genes, such as haemoglobin S, haemoglobin C, -alpha 3.7 deletional thalassaemia, G6PDA-, and HLA B*5301 [7]. However, Fulani have been shown to have higher levels of antibodies against two *P. falciparum* antigens [3] and stronger malaria-specific IgG, IgG1, IgG3 and IgM responses [8].

Further investigations into immunological differences that may affect susceptibility to *P. falciparum* have centred on FcγRIIa genotypes that affect the binding of different IgG subclasses. The FcγRIIa-H/H131 genotype is associated with higher levels of anti-malarial IgG2 and IgG3 antibodies, while the FcγRIIa-R/R131 genotype is associated with higher levels of IgG1 antibodies [9,10]. Individuals with the R allele of FcγRIIa have higher antibody levels than those with the H allele [11]. The FcγRIIa-H/H131 genotype and H131 allele are reported to exist at higher frequencies in the Fulani ethnic group [10]. However, there is a similar distribution of FcγRIIa-R131H polymorphism in Fulani and Mossi [11]. Overall, Fulani have higher antibody levels than Mossi [11]. Additionally, in Fulani, it is known that the IL4-524 T allele, which codes for the Th2 cytokine interleukin-4, is at high frequency and is associated with elevated antibody levels against malarial antigens [12]. A functional deficit of T regulatory cells may also contribute to Fulani having a higher resistance to malaria [4].

It has also been demonstrated that parasitaemia in asymptomatic Fulani is more common in individuals with lactase non-persistence genotypes (lactose intolerance), although this difference was not statistically significant [5]. This suggests that the potential immunoprotective properties of dietary cow’s milk may contribute to the partial malaria resistance of Fulani, yet this requires further investigation [5].

This commentary describes differences in susceptibility to *P. falciparum* infection between Fulani and Mossi subjects in the above-mentioned study [6].

### Ethics section

The protocol and the informed consent form were reviewed and approved by the Institutional Review Board of the Centre National de Recherche et de Formation sur le Paludisme and by the National Ethical Committee for Health Research of Burkina Faso. Prior to study initiation, a community meeting was held in each of the selected clusters to discuss the study with the community. The freedom of each individual household and a household member to decide on participation was discussed to minimize the potential influence of key opinion leaders in each cluster. Individual informed consent was obtained from each participant during a visit to the household before any study procedure.

## Results

### Baseline characteristics

In total, 14,075 subjects aged between one month and 104 years were enrolled. The intervention arm included 6,817 subjects with a mean age of 24.1 years, of whom 46.4% were male, 90.6% were Mossi and 8.7% were Fulani. The control arm included 7,258 subjects with a mean age of 23.4 years, of whom 47.4% were male, 95.7% were Mossi and 3.8% were Fulani. The intervention and control arms had similar proportions of children less than five years of age (17.0% and 15.6%, respectively).

### *Plasmodium falciparum* infection rates by ethnicity

#### Proportions of asymptomatic carriers

**Figure 1:**
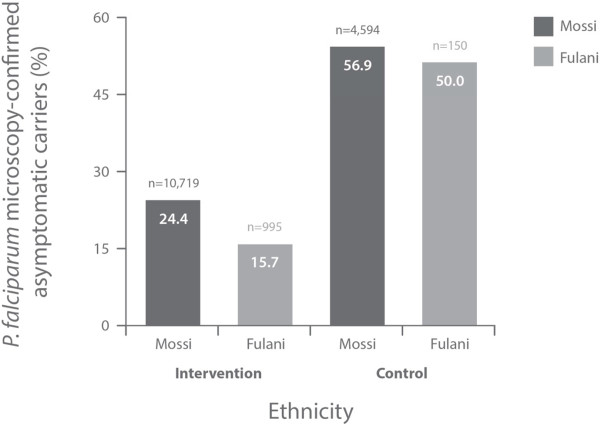
***P. falciparum *microscopy-confirmed asymptomatic carriers at any occasion.**

The Fulani groups had a lower proportion of microscopy-confirmed asymptomatic carriers than the Mossi groups at any occasion (Figure 1). Subjects may have been counted multiple times if diagnosed as asymptomatic carriers more than once during the study.

### Parasite densities (asexual forms and gametocytes)

**Figure 2:**
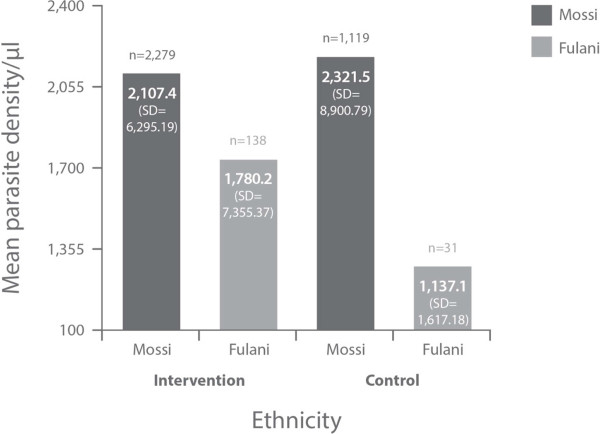
**Density of *P. falciparum *asexual forms by microscopy at baseline.**

**Figure 3:**
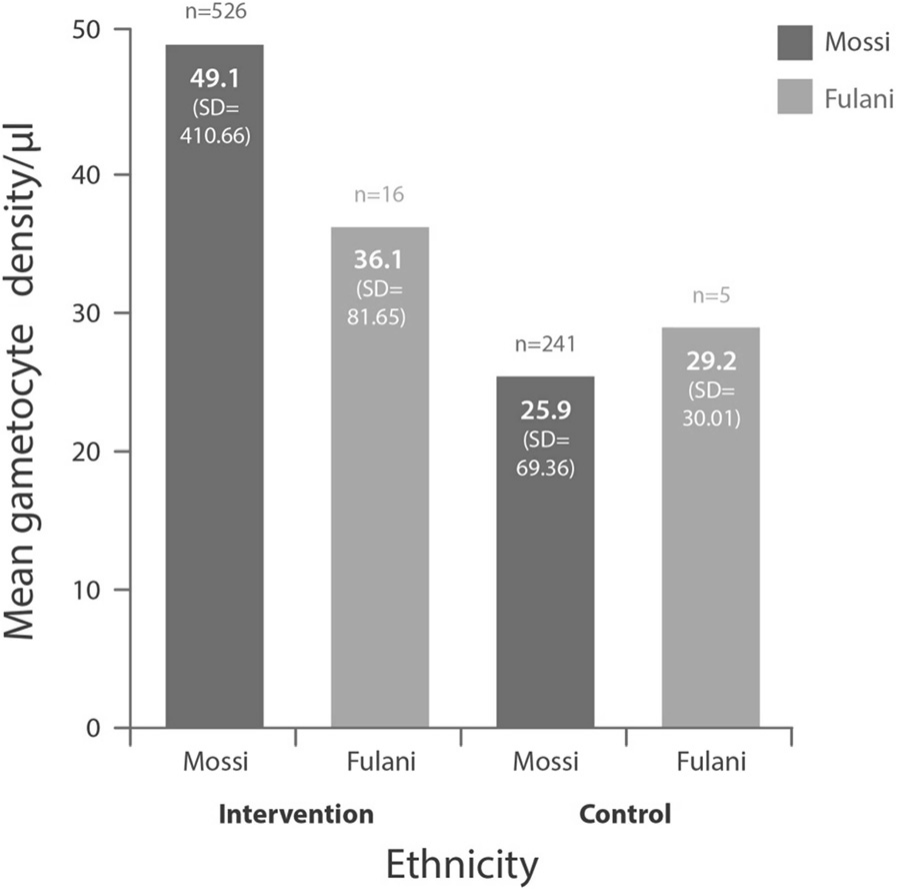
**Density of *P. falciparum *gametocytes by microscopy at baseline.**

The Fulani groups had lower densities of microscopy-confirmed *P. falciparum* asexual forms (Figure 2) and gametocytes (Figure 3) than the Mossi groups at baseline.

### Incidence of SMRC5000

In children under five years of age, the Fulani groups had lower rates of SMRC5000 per person-year than the Mossi groups at follow-up (Table 1). However, when the analysis included subjects of all ages, the Fulani groups had higher rates of SMRC5000 per person-year than the Mossi groups (Table 2).

**Table 1:** SMRC5000* per person-year in infants and children (under five years) at follow-**up (all subjects diagnosed at any time with asymptomatic carriage)**

**Study arm**	**Ethnicity**	**Number of SMRC5000**	**Person-year observed**	**SMRC5000 per person-year**
**Intervention (n = 512)**	Mossi	384	350.6	1.10
	Fulani	31	32.8	0.95
**Control (n = 203)**	Mossi	154	149.1	1.03
	Fulani	7	9.3	0.76

* SMRC5000 = Symptomatic malaria episodes including fever and a parasite density >5,000/μl.

**Table 2:** SMRC5000* per person-year in subjects of all ages at follow-**up (all subjects diagnosed at any time with asymptomatic carriage)**

**Study arm**	**Ethnicity**	**Number of SMRC5000**	**Person-year observed**	**SMRC5000 per person-year**
**Intervention (n = 2,740)**	Mossi	722	1928.1	0.37
	Fulani	57	116.8	0.49
**Control (n = 1,381)**	Mossi	257	1024.2	0.25
	Fulani	14	29.2	0.48

* SMRC5000 = Symptomatic malaria episodes including fever and a parasite density >5,000/μl.

## Discussion

Demographic surveillance data for the district show the distribution of Fulani and Mossi in the area as 7.1% and 90.7%, respectively. The proportions of each group enrolled in the study are generally consistent with this (8.7% and 90.6% in the intervention arm, 3.8% and 95.7% in the control arm).

The data we present confirm previously reported differences in susceptibility to *P. falciparum* malaria between Fulani and Mossi ethnic groups [1-5]. There was a consistently reduced incidence of the indicators for *P. falciparum* infection in Fulani over Mossi (Figures 1, 2 and 3 and Table 1), except for a reversal of this trend in the rates of SMRC5000 per person-year in subjects of all ages at follow-up (all subjects diagnosed at any time for asymptomatic carriage, Table 2).

This inconsistency in SMRC5000 per person-year in infants and children (under five years) and in subjects of all ages at follow-up (Tables 1 and 2) was unanticipated. One possible reason for this may be that treatment of asymptomatic carriers with artemisinin-based therapy may increase subsequent susceptibility to symptomatic malaria more in adult Fulani who are known to have stronger immune responses to malaria than in Mossi.

Therefore, areas for further research include a more detailed assessment of differences in SMRC5000 (per person-year) between children and adults and any biological reasons for these differences, as well as investigations into potential interethnic differences in the effect of artemisinin derivatives on naturally acquired protective immunity. Other beneficial studies may assess intra-Fulani susceptibility distribution, which may correlate with previously reported differences in anti-malarial antibody levels amongst the Fulani themselves [12].

## Conclusion

Data from the systematic, community-wide screening and treatment of asymptomatic carriers of *P. falciparum* with artemether-lumefantrine (AL) [6] confirm previously reported differences in *P. falciparum* susceptibility between Fulani and Mossi.

## Abbreviations

SMRC5000: Symptomatic malaria episodes including fever and a parasite density >5,000/μl.

## Competing interests

AT has received honoraria from Novartis Pharma AG, Basel, Switzerland to attend Advisory Board meetings to discuss this study. KH is an employee of Novartis Pharmaceuticals Corporation. SS has declared no competing interests.

## Authors’ contributions

All authors were involved in the design of the primary study and defining the content for, writing and critically reviewing this manuscript. AT and SS were involved in data collection, while AT and KH conducted the data analysis. All authors had full access to data in the study, discussed the results, reviewed the draft manuscript and agreed on the final version. AT had final responsibility for the decision to submit the manuscript for publication. Editorial assistance was provided by PreScript Communications, with funding from Novartis Pharma AG.
